# Prospective Preference Assessment for the Psilocybin for Enhanced Analgesia in Chronic nEuropathic PAIN (PEACE-PAIN) Trial

**DOI:** 10.1080/24740527.2024.2406285

**Published:** 2024-11-08

**Authors:** Jiwon Lee, Kaylyssa Philip, Duminda N. Wijeysundera, Hance Clarke, Cheryl Pritlove, Joel Katz, Paul Ritvo, Akash Goel, Muhammad Ishrat Husain, Karim S. Ladha

**Affiliations:** a Temerty Faculty of Medicine, University of Toronto, Toronto, Ontario, Canada; b Department of Anesthesia, St. Michael’s Hospital, Toronto, Ontario, Canada; c Department of Anesthesiology and Pain Medicine, University of Toronto, Toronto, Ontario, Canada; d Pain Research Unit, University Health Network, Toronto, Ontario, Canada; e Department of Anesthesia and Pain Management, University Health Network, Toronto, Ontario, Canada; f Li Ka Shing Knowledge Institute, Unity Health, Toronto, Ontario, Canada; g Dalla Lana School of Public Health, University of Toronto, Toronto, Ontario, Canada; h Department of Psychology, York University, Toronto, Ontario, Canada; i School of Kinesiology and Health Science, York University, Toronto, Ontario, Canada; j Campbell Family Mental Health Research Institute, Centre for Addiction and Mental Health, Toronto, Ontario, Canada; k Department of Psychiatry, University of Toronto, Toronto, Ontario, Canada

**Keywords:** Psilocybin, psychedelic, chronic neuropathic pain, prospective preference assessment

## Abstract

**Background:**

Negative perceptions of psilocybin and challenges of participant enrollment may represent barriers to conducting a randomized controlled trial examining psilocybin for chronic neuropathic pain.

**Aim:**

Prior to trial initiation, we aimed to examine patient attitudes toward the trial via a prospective preference assessment.

**Methods:**

Twenty-six patients with chronic neuropathic pain participated in a prospective preference assessment comprising quantitative (survey) and qualitative (interview) components. Content analysis was used to inductively and deductively identify factors that would motivate or discourage participation in the proposed trial. Demographics, clinical characteristics, and perceptions of psilocybin were collected to explore differences in characteristics between patients who were willing and unwilling to participate.

**Results:**

Survey results showed that most participants (76.9%) were willing to participate in the PEACE-PAIN trial. “Willing” participants reported higher prior psychedelic use (75%) as compared to the “maybe willing” (0%) and “not willing” participants (0%). Interviews indicated that the top two factors that motivated participation included the need for new treatment options (31.7%) and benefits to personal pain management (31.7%). The top two discouraging factors included practical difficulties of research participation (16.7%), and adverse events associated with psilocybin (16.7%).

**Conclusions:**

The PEACE-PAIN trial study design is supported by patient survey responses but may benefit from modifications, namely incorporating thorough discussions of the current evidence for efficacy, safety, tolerability, and approaches to address adverse effects of psilocybin. Additionally, the interest in participation by individuals with prior psychedelic use holds important methodological implications for the inclusion/exclusion criteria of the trial.

## RÉSUMÉ

**Contexte**: Les perceptions négatives de la psilocybine et les difficultés d'inscription des participants peuvent représenter des obstacles à la réalisation d'un essai contrôlé randomisé portant sur l’utilisation de la psilocybine pour le traitement de la douleur neuropathique chronique.

**Objectif**: Avant le début de l'essai, nous avons cherché à examiner les attitudes des patients à l'égard de l'essai par le biais d'une évaluation prospective des préférences.

**Méthodes**: Vingt-six patients souffrant de douleurs neuropathiques chroniques ont participé à une évaluation prospective de leurs préférences, comprenant des éléments quantitatifs (sondage) et qualitatifs (entretien). Une analyse de contenu a été utilisée pour déterminer de manière inductive et déductive les facteurs qui motiveraient ou décourageraient la participation à l'essai proposé. Les données démographiques, les caractéristiques cliniques et les perceptions de la psilocybine ont été recueillies afin d'explorer les différences de caractéristiques entre les patients disposés ou non à participer à l'essai.

**Résultats**: Les résultats du sondage ont montré que la plupart des participants (76,9 %) étaient disposés à participer à l'essai PEACE-PAIN. Les participants « disposés » ont rapporté une consommation antérieure de psychédéliques plus importante (75 %) que les participants « peut-être disposés » (0 %) et « non disposés » (0 %). Les entretiens ont indiqué que les deux principaux facteurs ayant motivé la participation étaient le besoin de nouvelles options thérapeutiques (31,7 %) et les avantages pour la prise en charge personnelle de la douleur (31,7 %). Les deux principaux facteurs de découragement comprenaient les difficultés pratiques de la participation à la recherche (16,7 %) et les effets indésirables associés à la psilocybine (16,7 %).

**Conclusion**: Le devis de l'essai PEACE-PAIN s’appuie sur les réponses des patients au sondage, mais pourrait bénéficier de modifications, notamment en incorporant des discussions approfondies sur les données probantes actuelles portant sur l'efficacité, l'innocuité, la tolérabilité et les approches pour traiter les effets indésirables de la psilocybine. En outre, l'intérêt pour la participation de personnes ayant déjà consommé des psychédéliques a des implications méthodologiques importantes pour les critères d'inclusion et d’exclusion de personnes de l’essai.

## Introduction

Chronic neuropathic pain is a debilitating condition characterized by shooting, burning, and stabbing sensations that are often constant and severe, leading to negative impacts on mood, sleep, and quality of life.^1^ Despite this, there is a paucity of therapeutic options for individuals with chronic neuropathic pain, with first-line agents demonstrating limited efficacy and poor tolerability.^2–4^ Thus, there is an urgent unmet need for novel treatments for chronic neuropathic pain. Preliminary evidence suggests the potential for psilocybin, the active component of “magic mushrooms,” to alleviate chronic pain.^5–10^ However, the therapeutic efficacy of psilocybin in chronic neuropathic pain remains understudied. To address this gap in evidence, we propose to conduct the Psilocybin for Enhanced Analgesia in Chronic nEuropathic PAIN (PEACE-PAIN) pilot, randomized, active-placebo controlled trial. Participants in the PEACE-PAIN trial will be adults, 18 years and older, with chronic neuropathic pain, and no self-reported improvement in symptoms to first-line medications recommended in the Canadian consensus guidelines (i.e., tricyclic antidepressants (TCAs), gabapentinoids, and serotonin noradrenaline reuptake inhibitors (SNRIs)).^11^ The effects of a single dose of psilocybin and concomitant psychotherapy on chronic neuropathic pain will be evaluated in comparison with the active placebo dextromethorphan and concomitant psychotherapy. Dextromethorphan was selected as the active placebo due to similar alterations in sensory perception, time to peak effect, and time-course as psilocybin.^12,13^

Prior to conducting the PEACE-PAIN trial, we sought to undertake a prospective preference assessment (PPA). PPA is a survey method by which investigators assess views of a proposed trial design and willingness to participate in the eventual study.^14^ Additionally, investigators can gather suggestions for improving the appeal of the study from potential trial participants through the PPA.^14^ In terms of the PEACE-PAIN trial, it was felt that the PPA may be particularly useful for enhancing patient acceptance and enrollment in the trial. While there has been a rapidly growing positive perception of psychedelics generally, and psilocybin particularly in treating various health conditions, a prior survey conducted in fibromyalgia patients indicated a negative perception of psychedelic drugs, including perceived associated health and legal status risks as controlled substances, that summated in concerns about participating in a psychedelic-based clinical trial.^15,16^ The negative views around psychedelic use may challenge patient acceptance of the PEACE-PAIN trial and, thereby, impede participant enrollment. This further adds to the existing concerns around clinical trials, which are generally challenged by issues of inadequate enrollment and selective enrollment that hinder successful trial completion and undermine the generalizability and validity of trial results. Conducting a PPA may facilitate potential modifications of the trial design, as well as mitigation of the barriers related to participant enrollment and worries around psychedelic use.

In this PPA, the objectives were to: (1) determine patients’ willingness to participate in the PEACE-PAIN trial; (2) identify areas for improvement in the trial protocol to enhance patient enrollment and acceptability; and (3) explore differences in characteristics between patients who would and would not be willing to participate in the PEACE-PAIN trial.

## Methods

### Setting and participants

A purposive sampling framework was used to recruit patients with chronic neuropathic pain from the chronic pain clinic at St. Michael’s Hospital.^17^ Recruitment took place during the month of August, 2023. A study team member approached potential participants and provided information about the study and the informed consent form. Informed consent was obtained from all participants prior to beginning the study. The inclusion criteria for the PPA were derived from the inclusion criteria for the PEACE-PAIN trial. Specifically, participants aged 18 years and older with chronic neuropathic pain (at least 3 months in duration) were included. The diagnosis of neuropathic pain was determined by a clinician with specialized training in chronic pain. Participants were excluded if they did not present with a working knowledge of the English language. This study was approved by the St. Michael’s Hospital Research Ethics Board (REB Study #23-133).

### Survey design

The PPA consisted of four sections: (1) A brief, researcher-produced vignette describing the proposed trial; (2) an assessment of the individuals’ understanding of the trial; (3) open-ended questions assessing attitudes toward the trial (i.e., factors that motivate and discourage participation); and (4) patient completed questionnaires. The vignette was verbally read to the participant by a researcher. A printed version of the vignette was also provided to the participant to follow along. Subsequently, the researchers assessed the participant’s understanding of the trial by asking the participants to answer four questions regarding the trial. Specifically, the patients were asked to answer whether the study will be blinded, the two types of study drugs, the study procedure concomitant to the study drug (psychotherapy), and how follow-ups are conducted. Following this, the participants were provided with open-ended questions for the interview. They were first asked “what factors might motivate you to participate in this type of study?” and, thereafter, “what concerns would you have if you were asked to participate in this study?” Finally, the participants completed a questionnaire, which included questions adapted from a previous survey on psychedelic use in chronic pain relating to severity of pain, pain medications used to date, effectiveness of current pain medications, interest in alternative pain treatments, previous use of psychedelics, prior knowledge of psilocybin, and perceptions of psilocybin.^11^ It also asked the patient to rate their willingness to participate in the PEACE-PAIN trial on a 6-point Likert scale: definitely not (1), probably not (2), maybe not (3), maybe (4), probably (5), and definitely (6), and provide demographics and clinical information, such as age, sex, years of education, visible minority status, previous research participation, and duration of illness. The vignette, questions assessing patients’ understanding of the trial, open-ended interview questions, and survey can be found in Supplementary File 1.

### Analysis

For the quantitative data, all statistical analyses were conducted using IBM Statistical Package for the Social Sciences (SPSS) Version 25. Descriptive statistics were used to examine the distribution of survey responses. Means and standard deviations were computed for continuous variables and percentages for categorical variables. Participants were grouped into “willing,” “maybe willing,” and “not willing” based on their responses to the 6-point Likert scale, where scores of 1–2 were considered “not willing,” 3–4 were considered “maybe willing,” and 5–6 were considered “willing” to participate.

Content analysis was conducted to examine the patterns from the patient interviews related to motivating and discouraging factors associated with the PEACE-PAIN trial.^18^ Dedoose data management software was used for coding, linking, and retrieving the qualitative data from the open-ended questions. Interviews were transcribed verbatim. Initial transcript sample readings were conducted independently by two researchers (KP and JL). During these readings, preliminary codes were identified. Differences were resolved and duplications were eliminated through conversation until consensus was reached by the two researchers. The final list of independent codes was sorted into categories inductively based on how the codes were related and linked.^19,20^ Subsequently, the categories were grouped under either one of the themes, motivating factors, or discouraging factors, through a deductive approach.^20^

For sample size, we decided a priori to calculate a minimum sample size prior to starting the study and to continue recruiting past the minimum sample size, as needed during data collection, until theoretical saturation was attained within the qualitative data. Hence, the final sample size of 26 resulted from, first, calculating a minimum sample size of 21 and then recruiting an additional 5 participants. The minimum sample size was determined by using an estimate of the proportion of patients who would be willing to participate in the trial, which was 70% based on a previous study.^15^ Using the estimate of 70%, in order to determine whether the lower bound of the 95% confidence interval is greater than 50%, the minimum number of patients needed in the PPA was calculated as 21 patients.

## Results

**Figure 1.**
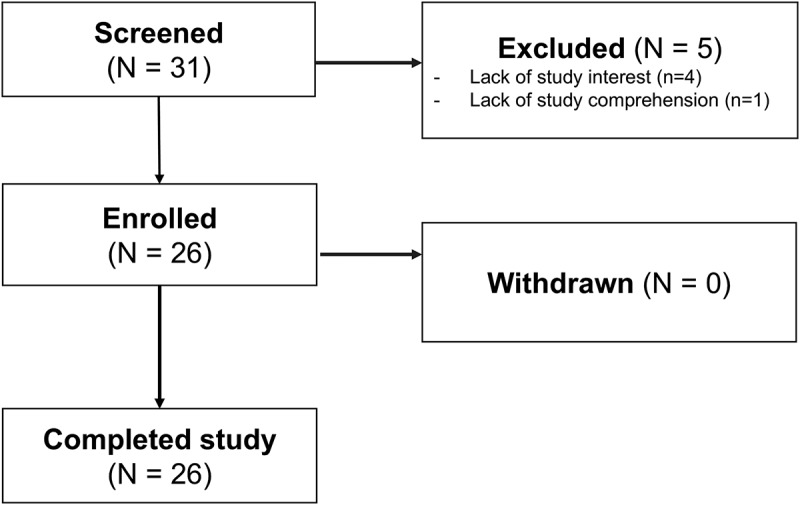
Patient enrollment flowchart.

Of the 31 participants who were screened, five participants did not enroll due to lack of study interest (*N* = 4) and lack of study comprehension (*N* = 1). Thus, 26 participants enrolled and completed the study (Figure 1). Overall, the mean age was 56.6 (*SD* 16.7) years, and the mean years of education was 15.5 (*SD* 2.9). Participants were predominantly female (*N* = 16, 61.5%), and a subset identified as a visible minority (*N* = 6, 24.0%).

In terms of clinical characteristics, the mean duration of chronic neuropathic pain was 162.4 (180.0) months, and the mean reported severity of chronic pain in the past month was 7.9 (1.6) on a scale of 0–10. The mean number of medications used for chronic pain to date was 6.5 (6.7), and the effectiveness of current treatments for managing chronic pain was rated as 2.4 (1.1) on a scale of 1 to 5. Regarding knowledge and perceptions of psilocybin, the mean self-reported degree of knowledge of psilocybin was 2.2 (1.1) on a scale of 1 to 5. The mean perceived potential of psilocybin as an effective treatment for chronic neuropathic pain was 3.4 (1.0), while the mean perceived risk of danger of psilocybin to health was 1.9 (0.8) on a scale of 1 to 5.

Based on responses to the 6-point Likert scale, 20 (76.9%) participants were “willing” to participate in the PEACE-PAIN trial, while 3 (11.5%) were “maybe willing,” and 3 (11.5%) participants were “not willing.” Fifteen (75.0%) participants in the “willing” to participate group had previously used psychedelics, of which 8 (40.0%) had used psilocybin specifically. In contrast, no participant reported prior psychedelic or psilocybin use in the “maybe willing” or “not willing” groups. Additionally, all participants (100.0%) were interested in alternative pain treatments, independent of their willingness to participate in the PEACE-PAIN trial. Full participant characteristics are detailed in Table 1.

**Table 1.** Characteristics of participants who were willing (*N* = 23), maybe willing (*N* = 3), and not willing (*N* = 3) to participate in the PEACE-PAIN trial.

Characteristic	All participants (*N* = 26), *N* (%) or mean ± *SD*	Willing (*N* = 20, 76.9%), N (%) or mean ± *SD*	Maybe Willing (*N* = 3, 11.5%), *N* (%) or mean ± *SD*	Not willing (*N* = 3, 11.5%), *N* (%) or mean ± *SD*
Demographics				
Age (years)	56.6 ± 16.7	52.9 ± 16.6	64.3 ± 6.0	73.7 ± 11.6
Sex				
Female	16 (61.5)	12 (60.0)	2 (66.7)	2 (66.7)
Male	10 (38.5)	8 (40.0)	1 (33.3)	1 (33.3)
Education (years)	15.5 ± 2.9	15.8 ± 2.8	12.3 ± 1.5	16.7 ± 3.1
Visible minority	6 (24.0)	4 (21.1)*	2 (66.7)	0 (0.0)
Previous research participation	6 (24.0)	4 (21.1)*	0 (0.0)	2 (66.7)
Prior psychedelic use	15 (57.7)	15 (75.0)	0 (0.0)	0 (0.0)
Prior psilocybin use	8 (30.8)	8 (40.0)	0 (0.0)	0 (0.0)
Clinical characteristics				
Duration of chronic neuropathic pain (months)	162.4 ± 180.0	118.8 ± 100.8	392.0 ± 313.2	224.0 ± 326.5
Severity of chronic pain in the past month (0 = no pain, 10 = worst pain imaginable)	7.9 ± 1.6	7.6 ± 1.6	8.3 ± 1.2	9.7 ± 0.6
Number of medications used to date	6.5 ± 6.7	7.4 ± 7.4	4.0 ± 1.0	3.3 ± 2.1
Interest in alternative pain treatments	25 (100.0)	20 (100.0)	3 (100.0)	2 (100.0)*
How effective are your current treatments in managing your pain? (1 = pain is not improving, 5 = pain is effectively managed)	2.4 ± 1.1	2.5 ± 1.1	3.0 ± 1.0	1.0 ± 1.0
Knowledge and perceptions of psilocybin				
How knowledgeable are you about psilocybin? (1 = not at all, 5 = extremely)	2.2 ± 1.1	2.2 ± 1.1	1.7 ± 1.2	2.67 ± 1.2
How much potential do you think psilocybin could have as an effective treatment for chronic neuropathic pain? (1 = none, 5 = extreme amount)	3.4 ± 1.0	3.7 ± 0.8*	3.0 ± 1.4	2.0 ± 1.0
How would you describe your perceptions of psilocybin in terms of their risk for danger to health? (1 = not at all dangerous, 5 = extremely dangerous)	1.9 ± 0.8	1.7 ± 0.7	2.3 ± 1.2	3.0 ± 0.0

*SD* = standard deviation. *Data was missing for one participant.

A total of 16 independent codes were identified from 26 participant interviews that revolved around themes of motivation (7) or discouragement (9) to participate in the PEACE-PAIN trial. Table 2 details the categories, analytical notes, and example quotes from the interviews grouped under motivating or discouraging factors. The need for new treatment options (31.7%) and benefits to personal pain management (31.7%) were the most frequent motivating factors. These were followed by patient knowledge and experience (13.3%), scientific advancement (13.3%), altruism (5.0%), other health benefits (3.3%), and trust of research (1.7%). The most common discouraging factors included practical difficulties of research participation (16.7%) and adverse events related to consuming psilocybin (16.7%). These were followed by lack of knowledge of study drugs (11.1%), lack of other study information (11.1%), negative perception of psilocybin (11.1%), general feelings of worry (11.1%), distrust of research (8.3%), patient specific health factors (8.3%), and aversion to randomization (5.6%). Further details are found in Table 3.

**Table 2.** Categories, analytical notes, and example quotes from patient interviews grouped under the themes of motivating or discouraging factors.

Categories	Analytical Note	Example Quotes
**Motivating**		
Altruism	Participants felt that the desire to help other patients through sharing their own experiences was a motivation to participating in the trial.	“I would like to contribute my own experiences, I think it would help people with chronic pain because it is extremely debilitating” *- willing, woman, 50*
Need for new treatment options	Participants expressed strong interests in trying new treatments for their pain, as they find that their current treatments are ineffective, have too many side effects, and negatively impact their daily life. Some participants explained that psilocybin, being a naturally derived medication, makes them more inclined to participate.	“Treatments for chronic pain has a lot of side effects and isn’t really effective and it would be nice to find a treatment that works” *- willing, woman, 22* “Interest in new ways of treating chronic pain.” *- not willing, woman, 72*
Benefit personal pain management	Participants felt that managing their pain was important, due to the severity, chronicity, and family history of their pain conditions, along with the desire for improved quality of life.	“If it helps with pain, absolutely” *- willing, man, 63* “To get rid of the pain in my back” - *maybe willing, woman, 54* “I don’t really have any motivation at this time although other than pain relief. So I guess it would be pain relief.” *- not willing, man, 63*
Other health benefits	Some participants described that they believe the trial participation may potentially help with their comorbidities and psychological well-being in addition to their pain.	“I’ve come to realize that there is a psychological component to my pain, which I had refused to accept [..] I’ve recently been told by my neurologist, maybe you have some components of OCD, maybe you have some components of anxiety, I had a bit of a rough childhood, and I have a very happy life other than my chronic pain, but maybe there is stuff in my past that needed to be dealt with that wasn’t dealt with. Is that, am I answering your question about the chronic pain part of it? Cause I feel like the psychological aspect of it is going to help me to assist me in my pain situation.” *- willing, woman, 52* “I have rheumatoid arthritis so it might help that too” *- maybe willing, woman, 65*
Patient knowledge and experience	Participants explained that they were open to participating in the trial based on what they have heard or prior experiences using psilocybin or psychedelics. They felt that the preexisting knowledge and experience makes them feel safer trying psilocybin and more optimistic about its analgesic properties. One participant appreciated the psychotherapy component of the trial, as they have heard that the psychological component of pain is also an important aspect to be addressed.	“what I’ve read and taking it myself, I find that it does really help and it helps to, like I said, calm me down or make me at least get my mind to focus on one thing, on mindfulness because my mind has so many things going on at once that I can’t calm it down. So that’s the reason why, that’s my background with the stuff, the psilocybin. I mean back in the days, obviously as a kid, we all use to joke around and take mushrooms and stuff right, but these aren’t the same things that we are talking about now. More refined and to the dose. So I’ve only tried recently, like I said, I’ve tried the capsules that someone has actually made. So they made 0.15 mg of it, and that was the dosage that I was taking, I took, for a couple days and it really really seemed to make me sleep and stay focused and actually get up and be able to do things. It worked for my pain.” *- willing, woman, 46*
Scientific advancement	Participants felt that the need for advancing the management of chronic pain was an important motivating factor to participating in the trial. They valued the need for assessing the effectiveness of psilocybin for chronic pain through scientific methods.	“Only one, contribution to medical science” *- willing, man, 80*
Trust of research	One participant felt that their trust in the researchers and hence the safety of the trial acts as a motivating factor to participating.	“Honestly I don’t really have any concerns about it because if it wasn’t safe, it wouldn’t be happening here, I trust these guys” *- willing, woman, 50*
**Discouraging**		
Aversion to randomization	One participant described an interest in receiving psilocybin and was concerned about the possibility of being randomized to the control group. Furthermore, they inquired about the possibility of receiving the effective medication following the completion of the study.	“I mean the only concern I have is to be in the control group obviously because obviously you want to get better, the one that will potentially make you better, but I know that it’s a study so that’s what happens. What I’d like is though that if I was in the control group, and if it’s possible, to afterwards get the one that actually does work – that would be my only concern.” *- willing, woman, 52*
Distrust of research	Some participants expressed concern about not being fully informed about all of the details of the trial and receiving sufficient support during the study. Another participant voiced concerns about being pressured to participate in the study.	“I would just like to have a good support behind me so that you know whatever, whoever is involved with it would give me all the information so that I would be well prepared.” *- willing, woman, 68* “it doesn’t say may be asked but it says will be asked to take one of the two study drugs, and they put the name. It appears as though even though you can.. you will be taken randomly or like flipping a coin, so meaning that my name could come up at anytime and I will, because of the word ”will ‘here, I will have to participate in either one of the two drugs and that’s my concern.’ - *maybe willing, woman, 58*
Lack of knowledge of study drugs	Participants described a lack of knowledge of psilocybin and dextromethorphan as concerns. They highlighted the need for additional details in order to make a decision about participating in the study.	“I don’t know anything about it” - *willing, man, 72* “I’m not sure what dextromethorphan is – I would need to know that” - *willing, woman, 73*
Lack of other study information	Some participants described the need for additional study details before making a decision about participating in the study. Specific details they requested include the exact dose that will be given, reason for psychotherapy, and feelings to expect after taking the study drug.	“how do you feel during it and after it and like all that kind of stuff ” - *willing, woman, 22* “It doesn’t say how much you are going to give each person” - *maybe willing, man, 70* “why do you have to take the psychotherapy? Why? What’s the reason for that?” - *maybe willing, woman, 58*
Negative perception of psilocybin	Some participants highlighted negative views associated with psilocybin as a deterring factor. In particular, concerns that were mentioned include the possibility of addiction, the poor reputation of psilocybin, association with illicit activity, and potential judgment by other people.	“I am an addictive personality and as such, I’ll expand on that, in that I’m a nonpracticing alcoholic and I am very careful what drugs I take because I don’t want to put myself back in that position.” - *willing, man, 72* “Magic mushrooms have always had such a bad rap, so it’s the worry of what it’s going to be like and a lot of people wouldn’t want to go on it, especially if you know you say you’re on magic mushroom and everybody has a fit, you know, so I’m not sure if I would even go on it” - *not willing, woman, 72*
Practical difficulties of research participation	Patients described concerns with the practical aspects of participating in a research study. They highlighted difficulties with having to attend follow up visits. They stated that it was particularly challenging having to attend multiple visits in-person. Furthermore, participants felt that it would take a significant amount of time to participate in the study, which could also interfere with their daily life. Finally, one participant mentioned that participating would simply be additional work that they would have to do to control their pain.	“Having to come in here a lot” - *willing, man, 80* “I think my first and foremost concern, would be, would I be able to work properly, while I’m participating in the study” - *willing, woman, 50* “It just seems that it would be one more thing that I have to kind of control, you know, during my day, which is already filled with controlling, trying to control pain. So it just seems that it would be adding to that.” - *not willing, man, 63*
Adverse events related to consuming psilocybin	Several participants described risks associated with psilocybin use and possible side effects as discouraging factors.	“There might be some side effects that might affect my work or my daily life.” - *willing, woman, 50* “the long-term risks of taking psilocybin” - *willing, woman, 28* “And the side effect is?” - *maybe willing, woman, 58*
General feelings of worry	Some patients described feelings of anxiety and uncertainty about what could happen as deterring factors.	“I guess just not knowing what would happen” - *willing, woman, 28* “It’s the worry of what it’s going to be like” - *not willing, woman, 72* “I’m not sure but I think that I would be anxious about it.” - *not willing, man, 63*
Patient-specific health factors	Several patients described specific health factors as discouraging factors. In particular, patients described potential conflicts with existing medications, possibility of a severe drug reaction based on a previous history of reactions, and the diverse impacts of the drug on each person.	“Only taking medications that might come in conflict with my transplant team.” - *willing, man, 80* “That I would have a reaction to it. I have a case of, the things that the doctors say should never react on me, they do react.” - *maybe willing, woman, 65* “So you give 1 person 50 mg another person 10, its all got different effects on people’s body.” - *maybe willing, man, 70*

**Table 3.** Frequencies of motivating and discouraging factors identified from patient interviews impacting willingness to participate in the PEACE-PAIN trial.

Categories	Willing, *N* (%)	Maybe willing, *N* (%)	Not willing, *N* (%)	Total, *N* (%)
Excerpts Pertaining to Motivating Factors (*N* = 60)				
Altruism	3 (5.0)	0 (0.0)	0 (0.0)	3 (5.0)
Need for new treatment options	18 (30.0)	0 (0.0)	1 (1.7)	19 (31.7)
Benefit personal pain management	15 (25.0)	2 (3.3)	2 (3.3)	19 (31.7)
Other health benefits	1 (1.7)	1 (1.7)	0 (0.0)	2 (3.3)
Patient knowledge and experience	8 (13.3)	0 (0.0)	0 (0.0)	8 (13.3)
Scientific advancement	8 (13.3)	0 (0.0)	0 (0.0)	8 (13.3)
Trust of research	1 (1.7)	0 (0.0)	0 (0.0)	1 (1.7)
Excerpts Pertaining to Discouraging Factors (*N* = 36)				
Aversion to randomization	2 (5.6)	0 (0.0)	0 (0.0)	2 (5.6)
Distrust of research	2 (5.6)	1 (2.8)	0 (0.0)	3 (8.3)
Lack of knowledge of study drugs	3 (8.3)	1 (2.8)	0 (0.0)	4 (11.1)
Lack of other study information	3 (8.3)	1 (2.8)	0 (0.0)	4 (11.1)
Negative perception of psilocybin	1 (2.8)	1 (2.8)	2 (5.6)	4 (11.1)
Practical difficulties of research participation	4 (11.1)	0 (0.0)	2 (5.6)	6 (16.7)
Adverse events related to consuming psilocybin	5 (13.9)	1 (2.8)	0 (0.0)	6 (16.7)
General feelings of worry	2 (5.6)	0 (0.0)	2 (5.6)	4 (11.1)
Patient-specific health factors	1 (2.8)	2 (5.6)	0 (0.0)	3 (8.3)

Of the 60 excerpts for motivating factors, 90.0% of responses were from the “willing” group, 5.0% of responses were from the “maybe willing” group, and 5.0% of responses were from the “not willing” group. Notably, altruism, patient knowledge and experience, scientific advancement, and trust of research were motivating factors unique to the “willing” group. Of the 36 excerpts for discouraging factors, 64.0% of responses were from the “willing” group, 19.6% of responses were from the “maybe willing” group, and 16.8% of responses were from the “not willing” group. Aversion to randomization was a discouraging factor only identified in the “willing” group.

## Discussion

Psilocybin is emerging as a potential treatment for chronic neuropathic pain,^5–10^ pointing to the need for clinical trials evaluating its safety and efficacy. Yet, negative perceptions specific to psilocybin along with the general challenges of participant enrollment may represent barriers to conducting such trials.^15^ As such, in this PPA study, we assessed the willingness of patients with chronic neuropathic pain to participate in the proposed PEACE-PAIN trial. We found that, of the 26 participants included in the study, 76.9% of patients were “willing” to participate, 11.5% were “maybe willing,” and 11.5% were “not willing.” Because most participants were “willing” to participate, the results suggest that the current study design of the PEACE-PAIN trial is considered acceptable by patients and that patient recruitment should be feasible.

Of the characteristics assessed, prior psychedelic use was a notable feature distinguishing participants who were “willing” to participate from those who were “maybe willing” and “not willing.” Specifically, the majority (75.0%) of “willing” participants reported prior psychedelic use, which accounts for 57.7% of the total participants assessed in this PPA, while none were reported in the “maybe willing” and “not willing” groups. The large proportion of prior psychedelic use among the “willing” participants holds important implications for the inclusion and exclusion criteria for the PEACE-PAIN trial. Specifically, excluding individuals with prior psychedelic use may be justified in a psychedelic-based trial, considering that prior psychedelic use may affect blinding and encourage selective enrollment of individuals with prior benefits from psychedelic therapy. However, our data indicates that excluding individuals with past psychedelic use may preclude inclusion of more than half of the patient population, as well as most of the participants who expressed willingness to participate in the trial. Consequently, this may negatively influence feasibility of the PEACE-PAIN trial by contributing to underenrollment. In fact, there appears to be a notable prevalence of psychedelic users among individuals with chronic pain in Canada. A North American survey in fibromyalgia patients reported that a lower, although still notable, proportion of patients (29.9%) had ever used a psychedelic; however, a minority of this sample represented patients residing in Canada (7.1%).^15^ A study examining chronic pain patients in Quebec found the prevalence of cannabis use for pain management to be 30.1%.^21^ While the lack of psychedelic studies in older individuals and chronic pain patients in Canada make it difficult to draw conclusions on the reasons for the high proportion of psychedelic use in this PPA, these prior surveys along with our findings of this PPA highlight the prevalence of psychedelic users in the Canadian population and the need to consider prior psychedelic use when designing a psychedelic-based trial. Furthermore, including individuals with a history of psychedelic use in the trial may still arguably be important, as these patients presumably used psychedelics without any concurrent psychotherapy. Namely, the PEACE-PAIN trial would provide an opportunity to explore the effects of combined modalities, even in patients who have prior psychedelic use. As such, how to incorporate prior psychedelic use in the inclusion/exclusion criteria (*e.g*., recency use as a factor) may be an important consideration in the design of the PEACE-PAIN trial.

Furthermore, the large proportion of prior psychedelic use among the “willing” participants as compared to the “maybe willing” and “not willing” groups may implicate prior psychedelic use as a potential predictor of willingness to participate in the PEACE-PAIN trial. Indeed, it has been previously suggested that individuals with prior psychedelic use may feel more comfortable with repeated use, including in the context of a psychedelic-based clinical trial, as well as be more optimistic toward the treatment potential of psilocybin for chronic pain based on personal experiences.^15^ In line with this, patient knowledge and experience with psychedelics emerged as one of the motivating factors for trial participation from the qualitative interview. Another explanation for the large difference in proportion of prior psychedelic use between the “willing” and “maybe willing”/“not willing” groups may relate to the stigma associated with psychedelics. Individuals without knowledge of and/or experience with psychedelics have previously demonstrated higher concerns around the legal and health implications of psychedelic drug use as compared to those who have previously used psychedelics.^15^ Consistent with this, judgment by others and the reputation of psilocybin were identified as concepts under negative perception of psilocybin as discouraging factors in the “not willing” group. This is quite relevant considering that all PPA participants (including those “maybe willing” and those “not willing”) expressed interest in alternative pain treatments, indicating that prior psychedelic use may, in part, influence the mismatch between the need for alternatives and the desire to participate in the PEACE-PAIN trial. Altogether, the difference in proportions of prior psychedelic use amongst the “willing,” “maybe willing,” and “not willing” groups highlights the need to address the concerns related to psilocybin perceptions to improve patient acceptance and enrollment in the PEACE-PAIN trial.

The analysis of patient interviews revealed three factors among those “not willing” to participate, categorized within the theme “discouraging.” These included: (1) negative perception of psilocybin (*e.g*., addiction, reputation of psilocybin); (2) practical difficulties of research participation (*e.g*., interference with daily life, too many study visits); and (3) general feelings of worry. These results are consistent with findings identified in a prior survey conducted in patients with fibromyalgia, which indicated that the negative perception of psychedelic drugs, such as the health risks and legal status of psychedelics, as well as financial, work, or transportation related limitations represented concerns to participating in a psychedelic-based clinical trial.^15^ To reassure the PEACE-PAIN study participants, a thorough discussion related to existing knowledge on the efficacy, tolerability, approaches to address adverse effects, and safety of psilocybin will be completed. This may help mitigate misconceptions and feelings of worry about psilocybin, which in turn, could encourage study participation.

Although we were unable to assess differences in characteristics between the groups, few characteristics appeared to be potentially related to willingness to participate. For instance, younger age, greater self-reported severity of chronic pain in the past month, and higher number of medications used to date appeared to relate with increased willingness to participate. Additionally, the “nonwilling” group appeared to have the highest self-reported knowledge of psychedelics despite lack of prior psychedelic use. It may be worthwhile to explore these potential relations further in future studies with a larger sample size, as well as in the PEACE-PAIN trial.

Several limitations should be noted. Firstly, the sample size of the “maybe willing” and “not willing” groups were small compared to the “willing” group, precluding us from conducting statistical analyses to assess differences between the groups. The small sample size of these groups may also undermine the internal validity of the findings around differences in prior psychedelic use between the groups. The small sample size of the “maybe willing” and “not willing” groups may, in part, have resulted from selective enrollment, where individuals who were not interested in the PEACE-PAIN trial or research in general were less willing to participate in the PPA in the first place. Indeed, most individuals who declined participation in the PPA stated lack of interest as the reason for not participating. Along similar lines, while we found the high proportion of prior psychedelic use as a distinguishing feature of willing participants, this may in part be attributed to selection bias, where those with prior psychedelic use were more willing to participate in the PPA. However, it is also possible that the individuals who initially declined participation perhaps reflect a less severe subset of chronic neuropathic pain patients, who are less interested in alternative pain treatments. Another limitation may be the small overall sample population, which may limit the generalizability of the findings. Nonetheless, the results still inform the PEACE-PAIN trial, as we reached theoretical saturation when conducting the participant interviews.^22^ Additionally, despite the small sample size, the sample had a mean age greater than 55 years and included a larger proportion of females, which reflects the demographic characteristics of the chronic pain population.^23,24^ Thus, the demographics captured by the study may support the generalizability of the study results to a larger patient population. Further, collecting information such as specific neuropathic pain conditions, specific psychedelics used, and the number of psychedelics used may have been helpful in better characterizing demographic differences between the groups. Moreover, although the survey covered several questions that aimed to understand patients’ experiences and views around psilocybin, it lacked questions to gauge experiences and views on psychotherapy. Hence, the findings may not provide insight into patients’ interest or disinterest in a trial involving psychotherapy and whether potential modifications can be made around the psychotherapy aspect of the trial intervention. Finally, although not necessarily a limitation of the present study, an important factor involved in studies of this nature that ask patients about their intentions or willingness to participate is the intention-behavior gap.^25^ In the present instance, there may be several reasons why the 77% who endorsed a willingness to enroll in the PEACE-PAIN might not follow through. For example, these patients may have been in an especially painful period when they initially endorsed a willingness to participate in the trial but may be experiencing less severe pain when the PEACE-PAIN trial begins. Factors such as time commitment and travel may additionally act as barriers to participating.

## Conclusion

Collectively, we demonstrated that 76.9% of potential participants were willing to participate in the PEACE-PAIN trial. A notable distinguishing feature includes the higher proportion of prior psychedelic use in the “willing” group compared to the “maybe willing” and “not willing” groups. Factors that discourage participation among individuals who were “not willing” to participate included the negative perception of psilocybin, practical difficulties of research participation, and general feelings of worry. A potential modification to the trial includes incorporating a thorough discussion of the current evidence for the efficacy, safety, tolerability, and approaches to address adverse effects of psilocybin. The findings of this PPA, particularly the interest in participation by individuals with prior psychedelic use, may have implications beyond the PEACE-PAIN trial, as it can be used to inform other psilocybin trials.

## Supplementary Material

Supplementary File 1.pdf

Revised Manuscript 2_clean_PPA_Canadian Journal of Pain_tracked changes.docx
