# Supplementary material for: Prospective Preference Assessment for the Psilocybin for Enhanced Analgesia in Chronic nEuropathic PAIN (PEACE-PAIN) Trial
Source: Can J Pain. 2024 Nov 8;8(1):2406285. doi: 10.1080/24740527.2024.2406285 (PMC11552286; doi:10.1080/24740527.2024.2406285)
Supplement: Supplementary File 1.pdf [file UCJP_A_2406285_SM2352.pdf]

Survey

Description of study (to be read to the patient):

Researchers at St. Michael's Hospital are studying new treatments for chronic neuropathic pain. This future study would like to test a study drug called psilocybin. Psilocybin is the chemical component of "magic mushrooms". Some studies suggest that psilocybin may be helpful in managing chronic neuropathic pain. It is important to study new drugs like psilocybin, because current medications for chronic neuropathic pain are not very effective and have side effects that are difficult to manage. Therefore, we are looking to find patients such as yourself to participate in a study testing psilocybin for chronic neuropathic pain.

In this future study, patients will be asked to take one of two study drugs, psilocybin or dextromethorphan, on a single occasion. Dextromethorphan is a study drug that produces the same feelings and experience of taking psilocybin. The two study drugs will look exactly identical to each other, and neither patients nor the researchers will know which study drugs were given. The type of study drug a patient receives will be random, like flipping a coin.

In addition to the study drug, participants will be provided with psychotherapy sessions. This is because psychotherapy is believed to help improve the effects of psilocybin. The sessions will occur over a 15 day period, once before taking the study drug and three times after taking the study drug.

Someone from the research team will call patients 1 month and 3 months after taking the study drug to see how well controlled their pain is.

Questions assessing patients understanding of the trial:

1) Can you tell me whether patients in the trial will be able to choose or know which study drug they are given?
2) Can you tell me what the two types of study drugs are?
3) In addition to taking study drugs, what other study procedure is involved in the study?
4) How will research staff contact participants to follow-up with them after taking the study drug?

Open-ended questions about factors influencing enrollment decisions:

1) What factors might motivate you to participate in this type of study?
2) What concerns would you have if you were asked to participate in this study?

Questionnaire

To be completed by the patient

Considering this information, how willing would you be to participate in this study? Please use the scale from 1 to 6, where 1 means you would definitely not participate, and 6 means you definitely would participate.

1	2	3	4	5	6
Definitely Not	Probably Not	Maybe Not	Maybe	Probably	Definitely

1) What year were you born? _______

2) Sex: ☐ Male ☐ Female

3) Do you consider yourself to be a visible minority?
☐ Yes ☐ No

4) How many years of school have you completed? Please circle one number.

1 2 3 4 5 6 7 8	9 10 11 12	13 14 15 16	17 18 19 20+
Grade School	High School	Post-Secondary	Graduate School

5) Have you ever participated in a research study before?
☐ Yes ☐ No ☐ Not Sure

6) How long have you had chronic neuropathic pain for? _______months / years

7) On average, how would you rate the severity of your chronic pain over the past month?

0 (No pain)	1	2	3	4	5	6	7	8	9	10 (Pain as bad as you can imagine)

8) How many medications have you used to date to manage your pain? _______

9) How effective are your current treatments in managing your pain?
1: My pain is not improving with the treatments I am currently using
2: My pain is occasionally managed with treatments I am currently using, but is mostly unmanageable
3: My pain is sometimes managed with treatments I am currently using, but is sometimes unmanageable

4: My pain is mostly managed with treatments I am currently using, but is occasionally unmanageable
5: My pain is effectively managed with the treatments I am currently using

10) Are you interested in exploring alternative treatment options for your pain?
☐ Yes ☐ No

11) Have you used psychedelics before? (e.g., psilocybin, magic mushrooms, LSD, DMT, mescaline, MDMA/ecstasy, Ketamine, Iboga/Ibogaine, Ayahuasca)
☐ Yes ☐ No

12) Have you used psilocybin before?
☐ Yes ☐ No

13) How knowledgeable are you about psilocybin?
1: Not at all knowledgeable
2: Slightly knowledgeable
3: Moderately knowledgeable
4: Very knowledgeable
5: Extremely knowledgeable

14) How much potential do you think psilocybin could have as an effective treatment for chronic neuropathic pain?
1: No potential
2: Slight potential
3: Moderate potential
4: Large potential
5: Extreme amount of potential

15) How would you describe your perceptions of psilocybin in terms of their risk for danger to health?
1: Not at all dangerous
2: Slightly dangerous
3: Moderately dangerous
4: Very dangerous
5: Extremely dangerous
